# Biomarker Characterization and Prediction of Virulence and Antibiotic Resistance from *Helicobacter pylori* Next Generation Sequencing Data

**DOI:** 10.3390/biom12050691

**Published:** 2022-05-11

**Authors:** Joana S. Vital, Luís Tanoeiro, Ricardo Lopes-Oliveira, Filipa F. Vale

**Affiliations:** Pathogen Genome Bioinformatics and Computational Biology, Research Institute for Medicines (iMed.ULisboa), Faculty of Pharmacy, Universidade de Lisboa, 1649-003 Lisboa, Portugal; joanavital@edu.ulisboa.pt (J.S.V.); luistanoeiro@gmail.com (L.T.); oliveira-ricardo@edu.ulisboa.pt (R.L.-O.)

**Keywords:** *H. pylori*, biomarkers, NGS, WGS, virulence, antibiotic resistance

## Abstract

The Gram-negative bacterium *Helicobacter pylori* colonizes c.a. 50% of human stomachs worldwide and is the major risk factor for gastric adenocarcinoma. Its high genetic variability makes it difficult to identify biomarkers of early stages of infection that can reliably predict its outcome. Moreover, the increasing antibiotic resistance found in *H. pylori* defies therapy, constituting a major human health problem. Here, we review *H. pylori* virulence factors and genes involved in antibiotic resistance, as well as the technologies currently used for their detection. Furthermore, we show that next generation sequencing may lead to faster characterization of virulence factors and prediction of the antibiotic resistance profile, thus contributing to personalized treatment and management of *H. pylori*-associated infections. With this new approach, more and permanent data will be generated at a lower cost, opening the future to new applications for *H. pylori* biomarker identification and antibiotic resistance prediction.

## 1. Introduction

*Helicobacter pylori* is a Gram-negative spiral-shaped stomach bacterium first discovered by Marshall and Warren in 1984 [1]. *H. pylori* is a strict human pathogen that traces human migrations, present in 50% of human stomachs worldwide [2], being associated with gastritis, peptide ulcer, mucosa-associated lymphoid tissue (MALT) lymphoma, and gastric adenocarcinoma [3,4]. Interestingly, increased disease complications, such as gastric carcinogenesis, may be associated with *H. pylori*-induced epigenetic changes [5,6]. Accordingly, *H. pylori* has been classified as a class I carcinogen by the International Agency for Research on Cancer since 1994 [7], being the strongest known risk factor for gastric adenocarcinoma. Notably, *H. pylori* infection can also lead to extra gastric diseases [8,9]. In 2017, *H. pylori* was included in the World Health Organization priority pathogens list for research and development of new antibiotics for its persistent resistance to clarithromycin treatment [10]. *H. pylori* strains show very high genetic diversity [11], which is the result of unusually high mutation [12,13] and homologous recombination rates [14,15]. Due to these intrinsic characteristics and the complex interaction of many factors, such as the host genetic background and environmental factors, the identification of biomarkers of early stages of infection has been challenging over the years. In this review, we will address the major *H. pylori* virulence factors and genes involved in antibiotic resistance, as well as the technologies currently used to detect them. We will show that the use of next generation sequencing (NGS) will lead to a faster characterization of virulence factors that can ultimately end in higher levels of personalized treatment and management of *H. pylori*-associated infections. NGS appears as a versatile technology able to provide answers to a multitude of questions, such as virulence factor determination, antibiotic resistance prediction, phylogenetic analysis, and epidemiologic follow-up, among others. Thus, it is anticipated that the use of NGS will continue to increase in the coming years.

## 2. Virulence Factors

*H. pylori* possesses several virulence factors that enable the bacterium to survive in an acidic environment, such as urease, and its movement and attachment to gastric epithelial cells, such as sheathed flagella and adhesins [16]. These virulence factors largely exceed the pure survival needs of *H.* *pylori*, allowing adjustment to the hostile milieu of the human stomach and the efficient maintenance of a persistent infection. Such features make *H. pylori* one of the most well-adapted human pathogens. Additionally, *H. pylori* possesses a variety of virulence genes that encode for effector proteins, which debilitate the gastric epithelium [17].

Although numerous bacterial genes have been defined to control *H. pylori* pathogenesis, there are two conventional virulence factors, the CagA protein, encoded in the *cag* (cytotoxin-associated genes) pathogenicity island (*cag*PAI) [18], and the VacA protein (vacuolating cytotoxin A) [19], which engage with various host molecules and trigger multiple downstream signalling cascades. Of note also is the role of some integrative and conjugative elements (ICEs) that have been found to be contributors to *H. pylori* virulence [20].

Importantly, the association of some of these factors with *H. pylori* pathogenicity appears to vary according to the geographical distribution of the *H. pylori* strains [21,22], revealing the impracticability of their use as global biomarkers and requiring regional procedures for disease evaluation and treatment.

### 2.1. Cytotoxin-Associated Gene Pathogenicity Island (cagPAI)

**Figure 1:**
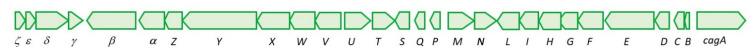
Schematic representation of *cag*PAI. The genetic arrangement of the 28 genes from the *cag*PAI of the reference strain *H. pylori* 26695 is depicted based on the organization proposed by Phuc et al., 2021 [26].

The *cag*PAI consists of a 40 kb region containing up to 32 genes (Figure 1) that encode for components of a bacterial type IV secretion system (T4SS). This system is involved in the translocation of an effector protein, CagA, into gastric epithelial cells, and in the host’s inflammatory response [23,24]. The *cag*PAI lowers GC content in comparison to the bacterial global GC content, suggesting that *cag*PAI was horizontally acquired. Most probably this horizontal acquisition occurred prior to human migrations out of Africa, since *cag*PAI can be found in geographically distinct populations throughout the world. [22]. Located at the 3′-end of the *cag*PAI region is the *cagA* gene, which encodes for the 120–145 kDa immunodominant protein CagA [25], which is undoubtedly the most studied *H. pylori* virulence gene.

The most recent models proposed for the Cag T4SS structure result from cryo-electron tomography studies [27,28]. In these models the Cag T4SS is formed by the outer membrane core complex (OMCC), which consists of a large mushroom-shaped structure with 14-fold symmetry, and an inner membrane complex (IMC), which consists of three concentric rings surrounding a central channel with 6-fold symmetry. The analysis of the OMCC structure was possible after successful extraction from *H. pylori*, revealing five main components: CagY, CagX, CagT, all of them with homology to OMCC components in other bacteria, and Cagδ and CagM, exclusive to *H. pylori* [29]. The OMCC assembly and stability seems to depend on CagY, CagX and CagM, whereas Cagδ and CagT appear to be localized in the periphery of the complex [28,29]. To date, CagA is the only protein known to be secreted by the Cag T4SS and translocated into host cells by adherent *H. pylori*. The detection of antibody responses to CagA was found to be more common in individuals with gastric cancer or peptic ulcer disease than in asymptomatic *H. pylori* positive individuals [30].

Although most *H. pylori* contain only a single copy of the *cagA* gene, some strains may contain multiple copies [31], confirming the high genomic variability characteristic of *H. pylori*, which can confer intermediate phenotypes. The CagA protein structure is defined by an organized N-terminal region and an intrinsically disorganized C-terminal region, both of which are required for the efficient secretion of CagA [32].

Throughout infection, CagA is placed on the plasma membrane, where it is phosphorylated by the host Src and Abl kinases at specific Glu-Pro-Ile-Tyr-Ala (EPIYA) motifs [33,34]. According to geography, there have been four different segments harboring EPIYA-motifs described, named as segments A, B, C and D, and the number and types of these EPIYA-motifs at the C-terminal region is related to CagA biological activity [25,35].

Several studies have shown that, after translocation, CagA interacts with multiple host cell molecules, leading to gastric epithelial hyperplasia, gastric polyps and adenocarcinoma of the stomach and small intestine, among others [36,37,38]. Due to this multiple evidence of a major role in gastric carcinogenesis, CagA has been designated the first bacterial oncoprotein [25,30,39].

Recent studies have shown a correlation between the presence of some *cag*PAI genes, such as *cagE*, *cagG* and *cagM*, and some tumors, suggesting these *H. pylori* genes as potential prognostic markers for gastric cancer [40].

The analysis of the geographical distribution of *cag*PAI showed its presence in more than 95% of *H. pylori* strains from western and south Africa and east and central Asia, 81% in northeastern Africa, an intermediate prevalence in strains from Europe and the Middle East, being detected in nearly half of the strains, and only 28% of strains from Latin America [22,41,42].

The *cagA* presence in the majority of East Asian strains, disregarding the disease status, disqualified it as a useful marker for the disease in this population. Interestingly, according to the EPIYA-motifs mosaicism, *cagA* positive strains can be grouped into west (EPIYA-ABC, EPIYA-ABCC and EPIYA-ABCCC) and east Asian strains (EPIYA-ABD) [43].

Since the use of the *cagA* gene as a global prognostic marker for the outcome of *H. pylori* infection is ruled out due to its almost universal presence in East Asian strains, distinct diagnostic procedures must be adjusted in different geographical regions [17].

The evaluation of the risk of development of gastric cancer in infected patients with *cagA* positive *H. pylori* strains must take account of its considerable global variation, along with the geographical incidence of gastric cancer [44]. While in western countries, a higher risk of gastric cancer and peptic ulcer disease development is correlated with the presence of *cagA*, in East Asia this correlation is less evident since the majority of *H. pylori* strains contain the gene [45].

The prevalence of *cagA* in children seems to occur in an identical manner to adults, varying geographically among different regions. CagA can be detected in more than half of *H. pylori* strains isolated from symptomatic cases in western countries [17,46,47,48], with the exception of Portuguese children that show a remarkably low prevalence of 22.4% [49]. As in adults, the high prevalence of the *cagA* gene in east Asian children has no clinical relevance [50,51], whereas in European children *cagA* was significantly associated with peptic ulcer disease, higher *H. pylori* density score and the degree of chronic and acute inflammation [47,52].

### 2.2. Vacuolating Cytotoxin A

The vacuolating cytotoxin A (VacA) is a relevant *H. pylori* pore-forming toxin secreted by a classical autotransporter pathway that plays a fundamental role in pathogenicity by interacting with gastric epithelial cells. The VacA name derives from its ability to induce vacuole formation in eucaryotic cells [53].

**Figure 2:**
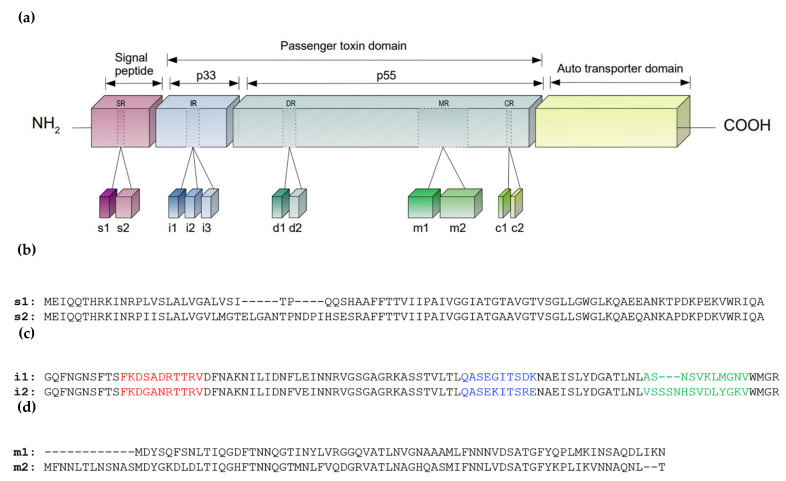
Schematic representation of the VacA protein and its variable regions. The different domains of the virulence factor are highlighted (**a**). Variable regions s (signal), i (intermediate), d (delete), m (medium) and c (c-regions) are represented along with their known variants. Based on Soyfoo et al., 2021 [56]. The amino acid residues of the main variable regions of VacA protein used for typing purposes are depicted (**b**–**d**). Sequence alignment of s1 and s2 regions (translated from sequence accession numbers LC187397 and LC187396, respectively) evidencing shorter s1 region (**b**); partial sequence alignment of i1 and i2 regions (translated from sequence accession numbers LC187485 and LC187502, respectively), evidencing cluster A (red), B (blue) and C (green). i1 and i2 classification is made based on sequence similarity with clusters B and C. Sequences displaying discordant pairing of the clusters B and C (one cluster i1 type and another i2 type) are classified as i3 (**c**), and sequence alignment of m1 and m2 regions (translated from sequence accession numbers EU551739 and EU551740, respectively) evidencing shorter m1 region (**d**).

Initially synthesized as a 140 kDa precursor that is secreted through a type V autotransport secretion system, the VacA protein is processed to yield two fragments, N-terminal p33 and C-terminal p55, that remain non-covalently associated and are thought to represent the protein functional domains (Figure 2). The p33 in the N-terminal is required for the formation of an inner channel for chloride transport [54], whereas the p55 in the C-terminal of protein is indispensable for binding of the toxin to host cells [53]. Both domains are needed for toxin oligomerisation [55].

Although all *H. pylori* strains carry the *vacA* gene, its polymorphisms lead to a considerable heterogeneity in the vacuolating activity phenotype, with only about 50% of the isolates displaying vacuolating activity [56]. This disparity between *vacA* strains is primarily attributed to *vacA* gene sequence variations (Figure 2) within the signal (s1 and s2), middle (m1 and m2), and intermediate (i1 and i2) [57], and, more recently, the deletion (d1 and d2) and c-regions (c1 and c2) [58].

VacA s2 toxins are produced and secreted at lower rates and are unable to form the membrane channels through which VacA s1 induces vacuolation of cells; therefore, the VacA s2 variant is considered less pathogenic than the s1 [19]. VacA i1 is also linked with increased vacuolating activity compared to VacA i2. VacA m1 induces a decrease in intracellular levels of glutathione and an increase in oxidative stress, leading to autophagy and apoptosis of host cells, contrary to VacA m2 [19].

Studies have shown that the combination of different sequences in these regions can determine the vacuolation ability [19]. Thus, the genotype with the combination s1/m1 exhibits high vacuolating activity, and the genotype s1/m2 has intermediate activity. Additionally, no vacuolating activity was shown by the genotype s2/m2 [59].

Gauthier et al. showed that the majority of the secreted VacA binds to cultured epithelial cells and uses lipid rafts as entry sites to be internalised by clathrinin-independent endocytosis [60]. Upon its incorporation by host cells, VacA accumulates inside different cellular compartments and can induce apoptosis. In addition, VacA can cause the dissipation of mitochondrial transmembrane potential, cytochrome C release, and activation of pro-apoptotic factor Bcl-2-associated X protein (Bax) by transferring to mitochondria, which results in apoptosis [61]. Moreover, VacA facilitates the persistence of *H. pylori*, inhibiting T-cell proliferation and effector functions by disrupting the epithelial cell tight junctions [62]. Even though VacA can interfere with the host inflammatory response by repressing T-cell activation, it also induces an inflammatory response generated by activation of NF-kB and results in upregulation of interleukine-8 (IL-8) [62].

Evidence that VacA triggers the endoplasmic reticulum stress response to activate the autophagy and increased cellular death of AGS cells has been shown [63]. In a meta-analysis study, Li et al. [64] observed an association between the VacA antibody and peptic ulcer disease and the risk of gastric cancer, and advocated the role of VacA as a biomarker predictor for these conditions.

The interaction between CagA and VacA, and how it can influence the outcome of *H. pylori* infection and gastrointestinal disease, had been widely shown before [24]. A recent study has demonstrated that VacA enhances bacterial survival independently of CagA accumulation [65]. Additionally, CagA can block the apoptotic activity of VacA, and VacA and CagA show antagonistic activities with respect to cellular morphology, with decrease in the number of vacuoles in elongated cells and reduction in protrusion length in cells displaying vacuoles [39].

### 2.3. Integrative and Conjugative Elements (ICEs)

*H. pylori* genomic islands were known as plasticity zones due to their variable gene content among strains. Presently, as a result of their capacity for conjugative transfer and recombination with the genome, they are characterized as integrative and conjugative elements (ICEs) [66]. These elements are conventionally designated as ICEHptfs3 and ICEHptfs4 [66], and include a T4SS distinct from the *cag*PAI, a subset of genes encoding DNA-processing (XerT recombinase involved in ICE excision/integration) and transfer function (VirD2 relaxase) enzymes and the characteristic sequence motifs that direct their activities [66,67,68,69]. Identical to *cag*PAI, tfs ICE possess all core genes of T4SS (*virB2*-*virB4*, *virB6*-*virB11*, and *virD4*). Each ICE island contains between 35 and 38 genes, 13 of which are conserved among all ICEs.

The nucleotide variety of ICEHptfs3 genes from *virB2* to *virB11* is highly similar among *H. pylori* strains, as shown by sequencing analysis. In contrast, tfs4 ICEs can be divided into three subtypes, 4a, 4b, and 4c, based on the sequence diversity of *virB2*, *virB3*, *virB4*, *virB6*, *virB7*, *virB8*, *virB11*, *virD2*, and *virD4* [26,66].

Delahay et al. [67] have shown that ICEHptfs3 is commonly found as fragments and is rarely complete, unlike ICEHptfs4. Moreover, ICEHptfs3 is more frequent in *H. pylori* within the HpAfrica1 population, suggesting that the presence of this element associates with a greater African genetic ancestry [67].

ICE element functionality has been determined in several studies by its capability to induce an inflammatory response in human gastric adenocarcinoma-derived cells (AGS), or to transfer selected genetic markers between *H. pylori* strains [69,70,71].

The ICEHptfs3 encodes the host-interacting pro-inflammatory cell translocating kinase (CtkA) protein [72] that stimulates host immune and epithelial cell proinflammatory signaling, suggesting it can potentiate gastric mucosal inflammation that might have consequences in inflammation-associated disease outcomes, such as atrophy and gastric cancer [73,74]. In fact, *ctkA* (encoded by jhp0940 in strain J99) has been identified as a marker for gastric cancer in some populations [75].

Importantly, and within ICEHtfs4, *dupA* is a duodenal-ulcer-promoting gene that encodes for the putative T4SS VirB4 ATPase [76]. The *dupA* gene is associated with increased levels of IL-8 in activated B cells [17]. In addition, the ATPase-associated efflux pump activity of the DupA protein, along with the activation of the mitochondria-dependent apoptotic pathway of the host’s cell, by inhibiting gastric cell growth, presumably confers its virulence [76,77,78].

The combination of *dupA* and other T4SS genes seems to be a more dependable marker for disease risk than the incomplete *dupA* cluster or *dupA* alone [79], indicating that tfs3 and tfs4b might form an alternative T4SS for DNA or effector protein in an identical way to *cag*PAI and can be considered virulence factors of *H. pylori* [26].

### 2.4. Other Virulence factors

There is a plethora of other virulence factors that contribute to *H. pylori*-induced pathogenesis, some of which are addressed below (Table 1).

#### 2.4.1. Urease

To survive the acidic gastric environment, *H. pylori* needs to reach the epithelium surface, surviving there between a pH of 5 and 6. The major protein involved in *H. pylori* resistance to acidity is the urease enzyme, which catalyzes the conversion of urea to ammonia and carbamate [80].

The urease operon is made of seven genes, arranged as *ureABIEFGH.* The urease enzyme forms a nickel-containing dodecamer composed of 12 UreA and 12 UreB subunits, with the help of the accessory proteins UreE, UreF, UreH and UreG [111,112]. Besides these proteins, the urease activity is promoted by acidic conditions, triggered by a proton-gated urea channel named UreI [113]. Furthermore, the urease gene expression is regulated by the acid responsive signaling regulon (ArsRS) and the nickel response regulator (NikR) [114,115,116].

#### 2.4.2. Flagella Chemotaxis System

The shape of the bacteria, which influences the ability to move in this environment, is maintained and produced by the activity of multiple enzyme networks that impact the peptidoglycan composition of the cell wall. *H. pylori* movement is often described as having a corkscrew-like manner to enable travel through the viscous mucus of the stomach lumen with the help of the flagella [117].

Each bacterium has several sheathed flagella composed of basal body, hook, and filament components [81]. The main structural proteins of the flagellum are HpaA, FlaA, FlaB, FliD and FlgK and most of their genes are not co-located or co-regulated on the chromosome [81,118]. There are more than 40 genes involved in the flagellar system, most are unclustered and transcription is controlled by different RNA polymerase sigma factors: σ28 (FliA), σ54 (RpoN) and σ80 [119].

Similar to other motile bacteria, *H. pylori* uses chemotaxis for spatial orientation [120]. Four methyl chemoreceptor proteins, TlpA, TlpB, TlpC and TlpD, sense external stimuli and repellents, forming a cascade reaction, first via CheW, that activates the histidine kinase CheA, which then phosphorylates CheY, the regulator responsible for the change in direction [81,119].

#### 2.4.3. Outer Membrane Proteins

Outer membrane proteins (OMPs) contribute to *H.* *pylori* virulence, suggesting some of these proteins as possible vaccine or drug targets. Importantly, *H. pylori* encodes five paralogous families of OMPs: (i) outer membrane porins (Hop) or Hop-related proteins (Hor), which code for porins (that are responsible for the transport of several molecules, including antibiotics by passive diffusion) and adhesins (that promote binding to epithelium cells, some of the more characterized members being SabA and BabA); (ii) iron-regulated outer-membrane proteins, which include the FecA and FrpB-like proteins (binding haem and haemoglobin); (iii) efflux pump outer membrane proteins; (iv) the Hof; (v) and Hom families (involved in adherence) [121,122].

##### AlpA/AlpB

AlpA and AlpB proteins, encoded by the operon *alpAB* are involved in adhesion to gastric epithelial cells by binding with laminin [96]. Studies using knockout mutants of *alpA* and *alpB* genes revealed a disadvantage in the *H. pylori* colonization capacity of mice, guinea pigs and Mongolian gerbils [96,123,124]. Additionally, AlpB is involved in biofilm formation [97].

##### BabA

BabA is the major adhesion protein of *H. pylori*. There are three genomic *loci* for *bab, babA*, *babB* and *babC.* Recent studies in *Rhesus macaques* showed a dynamic expression between *babA* and its paralog *babB*, with the overexpression of the latter resulting in a fitness advantage [125].

The main interaction of BabA is binding with the human fucosylated Lewis B antigen and the terminal fucose residues in antigen H, A and B in the gastric epithelium [126,127], triggering inflammation, development of intestinal metaplasia, and precancerous transformations [122]. An explanation of this seems to lie in the fact that the binding of BabA to Lewis B promotes the formation of the type IV secretion system (T4SS), enhancing the translocation of CagA through the gastric epithelial cells [125,126,128]. Furthermore, *H. pylori* strains positive for the *babA* gene, the *vacA-s1* type and *cagA* are more aggressive and induce severe inflammation, showing higher incidence of intestinal metaplasia when compared with strains only positive for the *vacA-s1* type and *cagA* [127,129].

##### HomB

HomB is the most characterized OMP from the Hom family [101,102,130,131]. There seems to be a correlation between the sequences of HomA, HomB and HomC and the geographic heterogeneity; HomD in contrast is highly conserved [101]. HomA and HomB share 90% homology when nucleotide sequences are compared [121].

The presence of *homB* has been considered a predictor of gastric cancer independent of *cagA* [130]. Further associations with other OMPs, such as BabA, accentuate the contribution to the hypervirulence of *homB*. Indeed, HomB has been proposed as a novel candidate marker for the development of peptic ulcer disease in children and young adults [52,103], promoting inflammation by association with increased secretion of IL-8 [52]. HomB seems not only to have a function in bacterial adherence, but also in biofilm formation, being necessary and sufficient to induce hyper-biofilm formation [101].

##### HopQ

HopQ belongs to the Hop family, with two types of alleles [121]. The type I allele is most common in East Asian *H. pylori* strains [132]. The outer membrane adhesin allows bacterial adhesion and delivery of CagA, by placing the T4SS-pilus at an adequate distance for delivery of CagA [104]. Indeed, HopQ is a co-factor of the T4SS, and the deletion of *hopQ* resulted in lesser T4SS-dependent activation signal pathways, such as NF-κB, MAPK signaling and IL-8 production [104].

##### OipA

Outer inflammatory protein A (OipA), also referred to as HopH, is regulated by a slipped-strand mechanism [133]. The role of this protein is controversial, but it seems to be involved in the adherence and proinflammatory response against the colonization of the gastric system, although no host receptor has been identified [133,134]. Though some studies in gastric cell lines have reported an increased production of IL-8 because of OipA [107,135], an in vivo study showed that OipA did not influence IL-8 production [136].

Furthermore, the role of OipA as an adhesion molecule is controversial as well. In fact, strains lacking the *oipA* gene have been claimed either to fail [137], or to succeed, in infecting animal models [138].

##### SabA

SabA is a sialic acid-binding adhesin, closely related to SabB, whose expression is regulated either by phase variation, with the same mechanism as OipA [139], or by a two-component signal transduction system, dependent on environment signals, such as increased pH and inflammation [135,140,141].

The main receptor of SabA is the Sialyl–Lewis X antigen, which is upregulated due to infection. So, the interaction is dynamic, inducing the expression of more Sialyl–Lewis antigens, which in turn strengthen bacterial adhesion to the epithelia contributing to the establishment of *H. pylori* colonization [109].

## 3. Genes Involved in Antibiotic Resistance

Antibiotic-resistant bacteria are one of the most important challenges for the world’s health and *H. pylori* is not an exception. For *H. pylori* eradication, generic guidelines suggest the use of a triple therapy, consisting of a proton pump inhibitor (PPI), and clarithromycin and amoxicillin as the first-line therapy, especially in contexts where clarithromycin resistance rates are predicted as moderate to low (<15–20%) [142]. Alternatively, a bismuth-based quadruple therapy involving a PPI, bismuth, tetracycline, and metronidazole, or a levofloxacin triple therapy consisting of a PPI, amoxicillin and levofloxacin are suggested as second-line eradicating therapies in contexts of high clarithromycin and low levofloxacin resistance levels, respectively [142]. Other salvage therapies have also been presented with their limitations and downsides [142]. Antibiotic-resistance is, indeed, the major obstacle to current eradicating treatments; the prevalence of antibiotic resistance in *H. pylori* was reviewed in detail for WHO regions [143]. The main antibiotics used for *H. pylori* eradication, the resistance mechanisms and the main resistance biomarkers are briefly reviewed below and are summarized in Table 2.

### 3.1. Clarithromycin

Clarithromycin is the most extensively used antibiotic for *H. pylori* eradication. It is a macrolide antibiotic, derived from erythromycin, that shows bacteriostatic activity by interfering with amino acid translocation, a key step in protein synthesis, upon binding the 50S bacterial ribosomal subunit [144]. Most primary resistance cases (c.a. 90% in western countries [144,171]) are reported as being due to point mutations on the V-domain of the 23S rRNA gene, decreasing the binding affinity of the large ribosomal subunit towards this antibiotic, thus reducing protein synthesis inhibition, and resulting in an antibiotic-resistant phenotype [144]. Although several point mutations have been reported with a wide range of impacts on clarithromycin resistance and should be evaluated [144,171], the most common resistance-associated mutations on the 23S rRNA gene are A2142G, A2142C and A2143G [144,172,173,174,175]. Apart from point mutations on the ribosomal RNA genes, other mechanisms seem to have a role in conferring resistance, or to at least potentiate the effect of 23S rRNA gene mutations on clarithromycin resistance. Mutations on *rpl22* (ribosomal protein L22) and *infB* (translation initiation factor IF-2) genes, both related to the translational apparatus, were suggested to be associated with clarithromycin-resistance [176]. Fitting a different strategy for antibiotic resistance, four efflux pump cluster candidates were identified in the *H. pylori* genome [177], and found to be associated with clarithromycin resistance [178].

### 3.2. Amoxicillin

Amoxicillin is a β-lactam antibiotic, similar to ampicillin, that acts, among other ways, by impairing the synthesis of peptidoglycan, namely by interfering with penicillin-binding proteins (PBPs), enzymes involved in the final stages of peptidoglycan synthesis and assembly [179]. Most cases of β-lactam resistance are depend on the expression of β-lactamases, β-lactam cleaving enzymes, and *H. pylori* is not an exception [180]. This, however, may not be the main mechanism for *H. pylori* amoxicillin resistance [171], which may instead be dependent on other mechanisms. The most important mechanism for amoxicillin resistance seems to be related to mutations on PBPs of *H. pylori*. Four PBPs have been described in *H. pylori*, three major (PBP1, 2 and 3) and one minor (PBP4) [181]. Mutations on the PBP1-coding gene, *pbp1*, resulting in C-terminal changes, are the most associated with amoxicillin resistance [158,182,183,184,185,186,187,188], although mutations on the remaining PBPs can also play a role [187,188]. Alternatively, mutations on the *hopB* and *hopC* genes, which encode outer membrane porin proteins, allow for amoxicillin diffusion out of the cell, leading to low intercellular levels of the antibiotic [171,183]. Other OMPs may also be involved and the potential role of efflux pump clusters for amoxicillin resistance is still unclear [171,182]. The range of mechanisms of resistance to amoxicillin presents a challenge when addressing *H. pylori* infection with this antibiotic.

### 3.3. Metronidazole

Metronidazole is an essential drug in the treatment of anaerobic infections that was developed to treat parasite infections [189,190] and is part of the suggested second-line eradicating therapy against *H. pylori* [142]. Metronidazole is delivered as a pro-drug that needs activation for further activity [191]. This activation is dependent on nitroreductase enzymes and requires a low redox potential, typical of anaerobes and possibly in *H. pylori* [171,191,192]. Reduced derivatives then lead to the inhibition of DNA synthesis and repair, resulting in bacterial death [191]. Although not indisputably, the microaerophilic requirements of *H. pylori* seem to allow for an additional mechanism of action involving a futile redox cycle with production of DNA damage-inducing reactive oxygen species (ROS) [191,192]. Resistance to metronidazole in *H. pylori* seems to depend on the intracellular redox potential [193] and it has long been associated with mutations on *rdxA* and *frxA* genes that encode an oxygen-insensitive NADPH nitroreductase and an NAD(P)H-flavin oxidoreductase, respectively [194,195]. Changes on *fdxB*, a gene encoding for a ferredoxin-like protein, was also suggested to be involved in metronidazole resistance [196]. Overexpression of the superoxide dismutase SodB, an essential enzyme for protection against ROS, was described as resulting from mutations on the ferric uptake regulator (Fur) which was demonstrated to impact metronidazole resistance in *H. pylori* [197,198]. Recently, temperature was shown to reduce metronidazole resistance in *H. pylori* [151]. In addition, a significant role of an efflux system was proposed, with a possible impact on other antibiotics as well [177,195,199].

### 3.4. Levofloxacin

Levofloxacin is a fluoroquinolone antibiotic showing both bactericidal and bacteriostatic effects that acts by binding to DNA gyrases and impairing their function [171]. These enzymes control topological transitions to the DNA structure and are critical in DNA replication, with such impairment resulting in organism death [171]. Similar to other species, *H. pylori* DNA gyrases are encoded by two genes, *gyrA* and *gyrB*. Resistance to levofloxacin relates to mutations in these genes, resulting in amino acid changes and rendering enzymes unaffected by the antibiotic. The most common mutations occur on the quinolone resistance-determining region (QRDR) of *gyrA* affecting codons 91 and 87 of the *gyrA* product, more specifically D91 to G [149,159,165,168], N [150,165,168,169,200] or Y [150,165,168] and N87K [150,159,165,168,201]. Despite this, changes in other codons of both *gyrA*, and, less frequently, *gyrB* products were, and continue to be, frequently reported, although the impact of some of them on antibiotic resistance is not yet well established [168,171,200]. Alternative mechanisms have not been described but were suggested to exist [165,168,171].

### 3.5. Tetracyclines

Tetracyclines are a group of antibiotics with special importance for *H. pylori* eradication in contexts of high clarithromycin resistance [142]. These antibiotics act by binding to the 30S ribosomal subunit, avoiding the attachment of the aminoacyl-tRNA to the ribosome A site, thus impairing protein synthesis [171,202]. The most important and well-known mechanism of tetracycline resistance is based on changes to the 16S rRNA sequence, mainly on positions 926 to 928 [203]. Interestingly, the level of resistance to tetracyclines seems to be proportional to the number of changes in the susceptibility-rendering AGA triplet [204]. Resistant strains with different mutations, or without mutations on the 16S rRNA gene, were reported, pointing to alternative mechanisms for tetracycline resistance [204]. Consistent with this, the inactivation of the putative efflux gene *hp1165* led to the loss of tetracycline resistance [205]; the impact of *H. pylori* cholesteryl-α-glucoside transferase on antibiotic-resistance to tetracyclines was also recently described [206]. In addition, mechanisms including roles played by efflux pumps, ribosomal protective proteins or oxireductases have been suggested [171].

The subject of *H. pylori* antibiotic resistance is of major importance and new mechanisms and new antimicrobial agents are subjects of continuous investigation [207,208,209]. Resistance to other antibiotics used in alternative *H. pylori*-eradicating therapies, such as rifabutin or streptomycin, have also been reported and are reviewed elsewhere [171]. Apart from specific molecular mechanisms for antibiotic resistance, the establishment of a biofilm has been suggested to render some degree of antibiotic resistance [210,211,212].

Increasing the knowledge of the main biomolecular markers of *H. pylori* antibiotic resistance can help in the rapid and cost-effective identification of the antibiotics to which the strains are resistant or susceptible. The assessment of antibiotic resistance/susceptibility profiles in *H. pylori* strains can aid dramatically in the choice of the eradicating therapy to use as first line on a case-by-case basis, diminishing the problem of persistent infection and secondary resistance events, and saving both time and resources.

## 4. Current Technologies Used for *H. pylori* Marker Detection

To a greater extent than identifying biomarkers for antibiotic-resistance profiling and virulence potential evaluation of *H. pylori* strains, the choosing of working methods to access such information in each context is of major importance. Currently, the most common molecular approach involves PCR amplification of the region (or regions) of interest, followed by Sanger-sequencing and contrasting of the obtained sequence with the genome of a reference strain, such as *H. pylori* 26695, or sequence databases.

For antibiotic-resistance in specific, phenotypic evaluation procedures, such as the agar dilution method—the gold standard—and E-tests are still widely used [213], and are often the starting point for the identification of resistance-conferring mutations [168,176,187]. However, these methods, apart from being time-consuming, costly and technically demanding, are widely subjective and culture-dependent. Several genotypic molecular techniques have been described to overcome the limitations of phenotypic techniques [214]. Accordingly, some PCR-based methods for *H. pylori* biomarker detection have been developed [215,216,217,218]. Of note, in 2009, Cambau and colleagues developed a PCR-based DNA strip genotyping test allowing for simultaneous detection of *H. pylori* and mutations predictive of clarithromycin and levofloxacin resistance [219]. Despite some limitations [220], the GenoType^®^ HelicoDR test is a simple and helpful test that produces better results when compared to histology- and culture-based methods [221].

With the advent of NGS technologies and the facilitated access to whole genome data, a novel perspective on *H. pylori* biomarkers and a range of new approaches to *H. pylori* study have been developed. A closer look at these new approaches is provided below.

## 5. New Approaches to *H. pylori* Biomarker Detection

**Figure 3:**
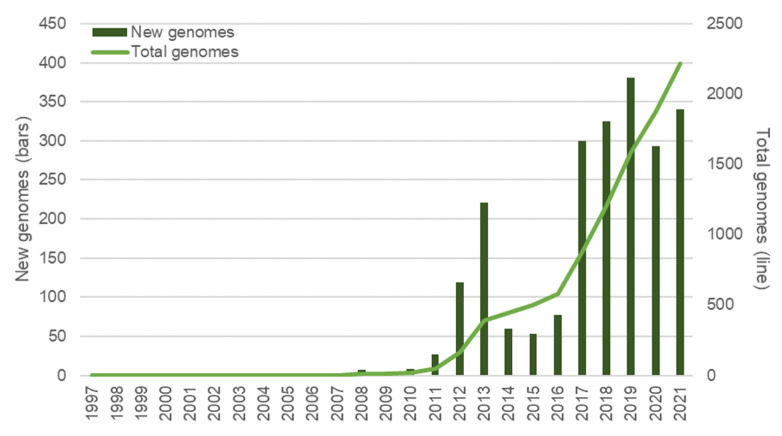
Overview of *H. pylori* genomes made available over time. Bars represent the new genomes released in each year; the total number of genomes available at each year is represented with a line. Data was retrieved from the PATRIC database [226] (last accessed in January 2022) and refer only to genomes submitted until the end of 2021; 34 genomes with no credible submission year were not taken into consideration for this graphic.

*H. pylori* was among the first bacterial species to have its genome fully sequenced [222] and was the first for which genomes of two independent isolates were sequenced and compared [223]. Twenty-five years later, the democratization of high throughput sequencing technologies that allow for the fast and massive acquisition of genomic data has cast a new light on the detection of *H. pylori* biomarkers related to both antibiotic resistance and virulence factors. As a result, the assessment of strain pathogenic capacity and the better choice of eradicating therapy is possible, saving precious time and resources in tackling this worldwide health problem (Figure 3). These new approaches enable several limitations of the previously referred to methods to be overcome and allow for a wider perspective on *H. pylori* biomarkers, pathogenicity and evolution. The importance and potential of whole genome data on bacterial pathogen evolution, virulence and pathogenicity has been acknowledged for over two decades [224] and we now have the technologies to get the best possible out of such data [225]. Presently (as of January 2022), a total of 2251 *H. pylori* genomes are recorded in the Pathosystem Resource Integration Center (PATRIC) database [226], reflecting the commitment of the scientific community to this technology (Figure 3). Curiously, the number of newly sequenced genomes diminished during the 2020 COVID-19 pandemic, with an apparent recovery in 2021 (Figure 3). The NGS systems mainly used for whole genome sequencing (WGS), as well as their advantages and disadvantages, have been reviewed elsewhere [227,228].

### 5.1. Sequencing Technologies

The most used sequence technology among studies assessing antibiotic resistance biomarkers or virulence factors was Illumina sequence-by-synthesis [26,71,144,229,230,231,232,233]. Interestingly, third-generation sequencing technologies, such as PacBio or Oxford Nanopore technologies, were used in either sole [170,234,235,236,237] or mixed approaches [238]. Different NGS approaches were also used, either for WGS [239,240,241,242], addressing, for instance, the correlation between genotypic markers and phenotype [243,244], or for amplicon sequencing, such as the *cag*PAI genes [245], or antibiotic-resistance biomarker genes [161]. In line with this, the accumulation of genomic data creates opportunities to reuse today’s data as new technologies, methods and perspectives arise.

Although most studies have extracted DNA for sequencing in a culture-dependent way, thus not overcoming this limitation, it is possible to obtain genomic data without culture [161]. Culture-independent whole-genome amplification, specifically targeting or not *H. pylori* sequences, was suggested to surpass the issue of the low amount of DNA retrieved from regular methods of acquisition [246]. Limiting the direct application of NGS technologies as an effective tool in combating *H. pylori*, some studies using whole genome data for the identification of virulence factors needed to use conventional PCR-based Sanger-sequencing to confirm the full sequence of genes [176,247,248,249,250]. As sequencing data increases in quality, confirmatory Sanger-sequencing is not also necessary and WGS data can be established as a culture-independent, objective, time- and cost-effective tool to obtain an integrated view of *H. pylori* genotypic characteristics and its pathogenic potential.

In recent years, American Molecular Laboratories (Vernon Hills, IL, USA) developed pyloriAR™/AmHPR^®^, an *H. pylori* antibiotic resistance NGS panel that targets the main genetic biomarkers associated with *H. pylori* antibiotic resistance [154,251], namely, the 23S rRNA gene (for clarithromycin), *gyrA* (fluoroquinolones), *rdxA* (metronidazole), *pbp1* (amoxicillin), the 16S rRNA gene (tetracycline) and *rpoB* (rifabutin). To our knowledge, a similar approach focusing on virulence factor-biomarkers does not yet exist, but seems developable in the near future, potentially increasing the range of options regarding the application of NGS technologies in assessing the pathogenic capacity of *H. pylori* strains.

### 5.2. Virulence Potential Evaluation and Antibiotic-Resistance Profiling

Whole genome data provide the opportunity to evaluate the virulence potential of strains and to predict the resistance profile for several antibiotics from a single dataset, with no need for multiple cultures, PCR amplification or sequencing procedures. Regarding virulence factors, several studies have used whole genome data to establish an association between virulence factors and the pathogenic effect of strains, and to determine the pathogenic potential of *H. pylori* strains. The confirmation of virulence phenotype upon biomarker detection, however, is not simple due to the previously discussed complex relationship between virulence factors and phenotypic outcomes. Conversely, regarding antibiotic-resistance markers, several studies have addressed the coherence between genomic-based predictions of antibiotic resistance (with previously approached biomarkers) and the phenotypic evaluation of antibiotic resistance. Most studies found a very strong correlation between 23S rRNA markers for resistance and resistant phenotype [154,157,170], especially with the detection of the A2143G mutation [232], although not indisputably [160]. Regarding levofloxacin resistance, the literature reports a moderate to strong reliability for the detection of mutations on the *gyrA* gene [154,170,243]. Mutations on the *pbp1* gene showed moderate to strong reliability for the prediction of amoxicillin resistance [154,155,160,252]. Less consistently, observations of metronidazole-resistance biomarkers have mostly been reported as fair predictors for metronidazole resistance [154,160,252], although some studies found only weak reliability for these observations [155,157,170]. Conversely, a good agreement between the truncated or mutated version of the *rdxA* gene and metronidazole-resistant phenotypes has been found [156]. A good correlation between the detection of 16S rRNA mutations and tetracycline resistance was also observed [160]. Furthermore, a close correlation between genotype data and phenotypic outcome has been observed [242]. Overall, existing studies suggest that genomic data can accurately predict antibiotic resistant phenotypes, especially for clarithromycin, levofloxacin and amoxicillin; however, it is necessary to highlight the importance of further research to uncover the complex phenotype-genotype correlation in *H. pylori* and to identify other antibiotic resistance mechanisms [242].

### 5.3. Current Tools as Possible Limiting Factors

When WGS data are obtained, efficient bioinformatics tools and representative databases are required to make the best use of such information in the characterization of antibiotic resistance. A wide set of sequencing-based tools for antimicrobial resistance detection and antimicrobial resistance reference databases have been reported and are available, as reviewed by Boolchandani and colleagues [253]. However, most of these tools are not particularly useful in the assessment of *H. pylori* resistance genotype, as resistance mechanisms in *H. pylori* are mainly based on point mutations, as previously noted, and these tools mainly focus on horizontally acquired genes [175]. Overcoming such limitations, the CRHP Finder webtool enables detection of most of the common mutations leading to clarithromycin resistance using *H. pylori* WGS data [254]. Additionally, the PointFinder tool recently integrated *H. pylori* information, allowing for the detection of point mutation-based resistance markers from WGS data [255]. Most studies, however, have compared the obtained sequences with the genome of the reference strains or susceptible strains, inferring sequence variations [161,167,176,240]. Regarding virulence factors, the ABRicate pipeline allows for the screening of genome contigs for virulence genes [26,238]. Since 2017, the Comprehensive Antibiotic Resistance Database (CARD) has been expanded and currently contains reference sequences of *H. pylori* factors of virulence and antibiotic resistance conferring mutations, namely on the 16S and 23S rRNA genes [256]. Interestingly, as previously noted, the accumulation of genomic data is a huge advantage, not only regarding statistical power, but also regarding the potential to reuse present data with future tools that have not yet been developed.

### 5.4. New Applications: The Example of Methylome Analysis

As new methods and new tools are developed, the application range of NGS technologies becomes even wider and more interesting, allowing for different approaches and the recovery of different information. One of the most interesting and promising applications of NGS methods is the assessment of methylome data. The methylome is the set of methylation modifications and their location in a particular genome, and is dependent on restriction-modification systems [257]. *H. pylori* present a wide range of restriction-modification systems and is densely methylated across the genome with methylation patterns differing widely between strains [258,259]. Importantly, genome methylation status seems to impact gene expression, including virulence factors [257]. Consistently, specific genome methylation was shown to play an important role for colonization and pathogenesis of *H. pylori* [260]. Interestingly, the single-molecule real-time (SMRT) sequencing developed by PacBio allows for the fast assessment of genome methylation status during sequencing-by-synthesis [228,257]. Complete methylome data and analysis were already reported for *H. pylori* strains [261,262]. The integrated analysis of methylome information, together with the previously discussed whole genome data, can thus be used to assess the pathogenic capacity of *H. pylori* strains. Again, with the natural development of new NGS technologies, new applications, such as methylome analysis, are anticipated, opening new perspectives for the integrated study of *H. pylori* genomic data.

## 6. Conclusions

The relevance of reliable biomarkers for a pathogen that is present in half of human stomachs worldwide and is classified as a class I carcinogen for its role in gastric adenocarcinoma has been demonstrated before and is addressed here. A variety of *H. pylori* virulence factors, some related to its adaptation to the harsh milieu of the human stomach, others directly involved in the infection process and pathogenicity, are important players in the complex mechanism of *H. pylori* interaction with the human host. The myriad of *H. pylori* virulence factors, their geographic variability and complex interplay, makes it difficult to ascertain the individual contribution of each one for *H. pylori* disease outcomes. Of importance, the *H. pylori* antibiotic resistance genes represent a major obstacle in identifying eradicating treatments. New technologies, such as NGS, provide more, increased quality, timeless genomic data that will enable several limitations of currently used methods to be overcome. The data from WGS offers a broader perspective on *H. pylori* biomarkers, permitting the establishment of an association between virulence factors and the pathogenic effect of strains, or creating the opportunity to predict the resistance profile for several antibiotics from a single dataset. The enormous amount of genomic data collected from these new technologies offers immense possibilities regarding its statistical power, but also for its storage and future reuse, as new tools for data processing and analysis emerge. The much faster identification and characterization of *H. pylori* virulence factors and antibiotic resistance genes, ultimately leading to early and personalized treatments of *H. pylori* infections, will arise in the future from the more frequent and extensive use of these technologies.

## Figures and Tables

**Table 1:** Main *H. pylori* virulence factors along with the respective interaction receptor and location, and suggested function.

Virulence Factor	Interaction Target	Target Location	Suggested Function	References
Urease	Urea	Gastric environment	Neutralize gastric acid	[80]
Flagella chemotaxis system	Not applied	Gastric environment	Bacterial movement to epithelial surface and deep gland	[81]
γ-glutamyl transpeptidase	Residues of Glutamine and Ammonia	T-cells, dendritic cells and epithelial cells	Adhesion, inhibition of T-cells, dendritic cells tolerization, apoptosis	[82,83,84,85,86]
Neutrophil-activating factor A	Unknown	Monocytes and dendritic cells	Induction of cytokines and TLR2 ligand	[87,88,89]
		Neutrophils	Chemotaxis and transendothelial migration of leukocytes	[90,91]
Tumor necrosis factor-α-inducing protein α	Nucleolin	Gastric epithelia	Induction of cytokines and chemokine and cell migration	[92,93,94,95]
AlpA	Laminin	Gastric epithelia	Adhesion	[96]
AlpB	Laminin	Gastric epithelia	Adhesion	[96]
	Unknown	Unknown	Biofilm formation	[97]
BabA	Lewis B blood group antigens	Gastric epithelia	Adhesion, CagA translocation via the T4SS	[98]
	Fucose residues on blood H antigen, A and B antigens salivary non-mucin glycoprotein	Gastric epithelia	Unknown	[99,100]
HomB	Unknown	Unknown	Biofilm formation, increase in IL-8 secretion, Adhesion	[101,102,103]
HopQ	Carcinoembryonic antigen–related cell adhesion molecule family (1,3,5,6)	Leukocytes/endothelial and epithelial cells	Adhesion, CagA translocation via the T4SS	[104,105]
HopZ	Unknown	Epithelial cells	Adhesion	[106]
OipA	Unknown	Unknown	Adhesion, induction of inflammatory cytokine production, apoptosis	[107,108]
SabA	Sialyl-Lewis X, Sialyl-Lewis A, Lewis X	Gastric epithelia	T4SS assembly	[109]
	Laminin (sialytaded moieties)	Gastric epithelia	Unknown	[110]
	Salivary glycoproteins (ex., heavy chain of secretory IgA1)	Saliva	Unknown	[99]

**Table 2:** List of consolidated biomarkers currently screened for antibiotic resistance profiling of *H. pylori* isolates.

Antibiotic	Main Resistance Mechanism	Associated Biomarker (Gene Product)	References *
clarithromycin	structural changes on antibiotic target	mutated 23S rRNA gene	[144,145,146,147,148]
		mutated *rpl22* gene (ribosomal protein L22)	[149,150]
		mutated *infB* gene (translation initiation factor IF-2)	[149,150]
metronidazole	inactivation/activity reduction of pro-drug activators	mutated *rdxA* gene (oxygen-insensitive NADPH nitroreductase)	[151,152,153,154,155]
		mutated *frxA* gene (NAD(P)H-flavin oxidoreductase)	[151,152,153,156,157]
amoxicillin	structural changes on antibiotic target	mutated *pbp1* gene (penicillin-binding protein 1)	[154,155,158,159,160]
tetracycline	structural changes on antibiotic target	mutated 16S rRNA gene	[155,161,162,163,164]
levofloxacin	structural changes on antibiotic target	mutated *gyrA* gene (DNA gyrase subunit A)	[146,152,165,166,167]
		mutated *gyrB* gene (DNA gyrase subunit B)	[156,159,168,169,170]

* Exemplificative studies targeting the mentioned biomarker are referred.
